# A New Insight on Cardoon: Exploring New Uses besides Cheese Making with a View to Zero Waste

**DOI:** 10.3390/foods9050564

**Published:** 2020-05-02

**Authors:** Cássia H. Barbosa, Mariana A. Andrade, Fernanda Vilarinho, Isabel Castanheira, Ana Luísa Fernando, Monica Rosa Loizzo, Ana Sanches Silva

**Affiliations:** 1 Department of Food and Nutrition, National Institute of Health Dr. Ricardo Jorge, Av. Padre Cruz, 1649-016 Lisbon, Portugal; cassia.barbosa@insa.min-saude.pt (C.H.B.); mariana.andrade@insa.min-saude.pt (M.A.A.); isabel.castanheira@insa.min-saude.pt (I.C.); 2 REQUIMTE/LAQV, Faculty of Pharmacy, University of Coimbra, Coimbra, Azinhaga de Santa Comba, 3000-548 Coimbra, Portugal; 3 MEtRICs, Departamento de Ciências e Tecnologia da Biomassa, Faculdade de Ciências e Tecnologia, FCT, Universidade Nova de Lisboa, Campus de Caparica, 2829-516 Caparica, Portugal; ala@fct.unl.pt; 4 Department of Pharmacy, Health and Nutritional Sciences, University of Calabria, 87036 Arcavacata di Rende, Italy; monica_rosa.loizzo@unical.it; 5 National Institute for Agricultural and Veterinary Research (INIAV), I.P., Rua dos Lagidos, Lugar da Madalena, Vairão, 4485-655 Vila do Conde, Portugal; anateress@gmail.com; 6 Center for Study in Animal Science (CECA), ICETA, University of Oporto, 4051-401 Oporto, Portugal

**Keywords:** *Cynara cardunculus* L., cardoon leaves, by-products, antioxidant activity, antimicrobial activity

## Abstract

Cardoon, *Cynara cardunculus* L., is a perennial plant whose flowers are used as vegetal rennet in cheese making. Cardoon is native from the Mediterranean area and is commonly used in the preparation of salads and soup dishes. Nowadays, cardoon is also being exploited for the production of energy, generating large amount of wastes, mainly leaves. These wastes are rich in bioactive compounds with important health benefits. The aim of this review is to highlight the main properties of cardoon leaves according to the current research and to explore its potential uses in different sectors, namely the food industry. Cardoon leaves are recognized to have potential health benefits. In fact, some studies indicated that cardoon leaves could have diuretic, hepato-protective, choleretic, hypocholesterolemic, anti-carcinogenic, and antibacterial properties. Most of these properties are due to excellent polyphenol profiles, with interesting antioxidant and antimicrobial activities. These findings indicate that cardoon leaves can have new potential uses in different sectors, such as cosmetics and the food industry; in particular, they can be used for the preparation of extracts to incorporate into active food packaging. In the future, these new uses of cardoon leaves will allow for zero waste of this crop.

## 1. Introduction

*Cynara cardunculus* L. is a perennial plant belonging to the family Asteraceae, which is native to the Mediterranean area [1,2,3,4,5]. Commonly known as cardoon or artichoke thistle, *Cynara cardunculus* L. is a complex species comprising three botanical varieties: the globe artichoke (var. *scolymus* (L.) Fiori), the cultivated cardoon (var. *altilis* DC.), and the wild cardoon (var. *sylvestris* (Lamk) Fiori) [5,6]. This review is focused on the cultivated and wild cardoons that herein are referred to as cardoon.

Cardoon is considered to be a valuable crop as it shows high yields, drought tolerance, and low input needs, and it provides benefits regarding soil properties, erodibility, and biological and landscape diversity [7]. The edible parts of cardoon are the fleshy stems and the immature heads, which, traditionally, are used in Mediterranean cuisine, mainly in salads and soup dishes [1,8]. In Italy, for example, the edible parts are used in many dishes as a vegetable and are sold canned in olive oil. Besides, they are also used in other Mediterranean traditional dishes such as Tunisian or Algerian “couscous”, and the flower (the pistils) is used in cheese making as a vegetable rennet substitute [4,9,10,11,12,13]. Cardoon by-products are mainly composed of leaves, stems, and seeds. They have been used to produce biomass for energy; and oil for human consumption, biodiesel, and animal feed [14,15]. The leaves are used in traditional medicine due to their high content in bioactive compounds such as cynarin and silymarin [2,4,14,15,16,17,18,19]. Moreover, the cultivation of cardoon in large areas is gaining interest as feedstock for novel industrial bio-based products (e.g., conversion into biopolymers or as a source of cellulose for nanometric technological applications) [20,21].

The world population is growing every day, and consequently, increasing the demand for food. In consequence, food production has been intensified, generating tons of by-products, which, if not discarded in a sustainable and responsible way, can represent a serious environmental problem, depending on the composition of the by-product itself. Despite its known powerful biological properties, unfortunately, the majority of these by-products are not being applied for other purposes, even though studies have announced their potential in different areas. For instance, several research studies in progress are incorporating fruit by-products in active food packages to delay the natural lipid oxidation phenomenon and microbial deterioration of foods [22,23,24]. Additionally, the increased demand for bioproducts, biomaterials, and bioenergy may result in a higher accumulation of by-products from the value chains. Therefore, this review aims to systematize the current knowledge of cardoon and emphasize the main composition of the by-products of the plant. Specifically, this review highlights the main bioactive compounds of cardoon leaves and their functional properties. Finally, their potential uses are also addressed, with a special focus on active food packaging to extend food shelf life.

## 2. Cardoon Botanical Description, Distribution, and Cultivation

Although native from the Mediterranean area (southern Europe and North Africa), cardoon has been spread to several other countries like the United States of America, Mexico, Australia, and New Zealand [1]. Due to its natural habitat, cardoon can grow in adverse climate conditions, with high temperatures, severe drought, and in thin unproductive and stony soils [1,14,25,26]. Moreover, cardoon is also a pollinator-supporting industrial crop, with all the associated benefits in terms of biodiversity [7,26,27,28].

Cardoon is a perennial plant that can grow up to two meters high with thick and rigid stems. It has an annual development cycle, and the reproductive cycle is completed by summer. With adequate soil moisture and temperature, cardoon development can start during autumn or spring with seed germination [5,14,29]. The seeds are light grey, brown, or black and can be up to 8 mm long [1]. Cardoon starts by developing a root that can grow one meter down and regenerates each year [1,5,14,29]. Simultaneously, the leaves grow to originate a leaf rosette. The leaf rosette is large and strong, with over 40 leaves that can be up to 120 by 30 cm, while the leaf from the upper stem can be up to 50 cm in length [1,5,14]. By late spring, the plant develops its inflorescence at the top of a branch on the stem, which can be up to 3 m high and about 2–4 cm of diameter. The inflorescence can be called capitula or heads and have an almost round shape. The cardoon inflorescence consists of several hermaphrodites and tubular flowers (florets) fitted in a well-developed receptacle. The florets are usually blue–violet colored [1,5,14,29]. By summertime, the aboveground plant parts dry, but the underground parts (the roots and perennating buds) remain alive until weather conditions become milder, and the perennating buds sprout, and a new development cycle starts [14,29].

## 3. Cardoon Flower—Cheese Making

**Figure 1:**
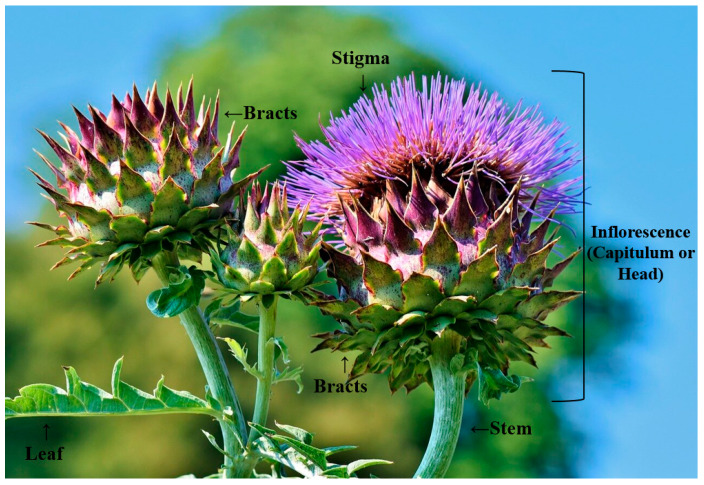
Cardoon flower [34].

Cardoon flowers (Figure 1) are used as milk clotting in cheese making, producing a cheese with a creamy soft texture and a genuine and slightly piquant aroma [12,30,31,32]. The clotting activity is due to the stigma and style of the inflorescence. Two proteases have been identified as responsible for this activity: cardosins A and B [12,31,32,33]. Both enzymes are responsible for the milk clotting and have proteolytic activities. Specifically, cardosin A is responsible for the clotting activities by the hydrolysis of the k-casein, and cardosin B is responsible for proteolysis, similar to pepsin activity [12,31].

Cardoon flower, as a coagulant agent for cheese making, is used mostly in Portugal, Spain, and Italy [13,30,31,32,33]. In these countries, some cheeses, classified as protected designation of origin (PDO), are produced using cardoon flower as a coagulant [11,30,32]. The PDO denomination is legally defined as a product that has a specific origin and/or production that takes place in a specific geographic area and endorses the quality and authenticity of the product [11,35]. For instance, for the “Serra da Estrela” cheese, a well-known Portuguese semi-soft cheese made with raw ewe’s milk of a native breed (Bordaleira da Serra da Estrela), the usage of cardoon flower is crucial for its authenticity and its high quality [30,36,37].

Gomes et al. [32] described a process to produce the cardoon flower extract to use as a natural rennet for cheese making. For this purpose, the flower is collected during the flower season. Then, the flower pistils are cut and separated from the rest of the plant and then dried under room temperature (23 ± 1 °C), protected from sunlight. After that, and according to the traditional method, the dried pistils are macerated by hand in a mortar with acetate buffer and then filtrated to a final volume of 50 mL at room temperature [30,32,33].

Moreover, since cardoon flower is used directly in dairy products, it is important to know its chemical composition, in particular, its content in phenolic compounds and its bioactive properties. Therefore, several authors have studied the cardoon flower composition. Dias et al. [36] characterized the phenolic composition of the inflorescence of cardoon flower and evaluated the antioxidant and antibacterial properties. The authors concluded that flavonoids are the major family of compounds present in cardoon flower; these include apigenin (9.9 ± 0.5 mg/g of extract) and caffeoylquinic acid (7.6 ± 0.2 mg/g of extract), which were the major compounds identified. Regarding the antibacterial activity, the cardoon flower presented a good outcome against Gram-positive bacteria such as *Listeria monocytogenes*, methicillin-sensitive *Staphylococcus aureus* (MSSA), and methicillin-resistant *Staphylococcus aureus* (MRSA); and Gram-negative bacteria such as *Morganella morganii* and *Pseudomonas aeruginosa*. Overall, the authors concluded that cardoon flowers, for use as rennet in cheese making, is a highly valuable ingredient as a source of bioactive compounds [36].

## 4. Cardoon By-Products and their Current Applications

### 4.1. Biomass

Cardoon crops are capable of growing in adverse environments, and they have been identified as potential crops for energy production [27]. Cardoon crop by-products are mainly used to produce biomass for different applications. At an industrial level, cardoon crops represent a great interest in the production of solid biofuel, seed oil, biodiesel, paper pulp, green forage, and pharmacologically active compounds [14,38,39,40,41,42,43]. Mauromicale et al. [27] studied the potential ability of cultivated and wild cardoons to produce energy in terms of biomass, achenes, and energy yield. The authors concluded that both cultivated and wild cardoon are potential energy crops and improved the soil fertility characteristics by increasing organic matter, total nitrogen, available phosphorus, and exchangeable potassium content. The annual average outcome of cultivated cardoon presented was 14.6 t/ha of dry biomass, 550 kg/ha of achenes, and 275 Gj/ha yields, while for wild cardoon, the outcome was 7.4 t/ha of dry biomass, 240 kg/ha of achenes, and 138 Gj/ha of energy yield. Several authors [43,44,45] studied cardoon for biomass production and demonstrated that this crop can be grown as an energy crop. These authors concluded that the cardoon aboveground dry biomass yield continuously increased during the seasons and that the low yield in the first season may be due to establishment difficulties, especially under the low input management in very dry conditions [27,43,44,45].

#### 4.1.1. Solid Biofuel

Cardoon production is mainly based on the production of biomass for the production of biofuel. The solid biofuel is generally used for heating applications and power generation [14,46]. This is mainly due to the biomass characteristics that have high productivity in severe climate conditions; it has low moisture content, and it has high lignocellulose content [14]. Several studies have been made to assess the characteristics of cardoon as a solid biofuel [14,29,47].

#### 4.1.2. Seed Oil and Biodiesel

Cardoon biomass can also be used to produce biodiesel and seed oil, which in turn can be used for human nutrition or biodiesel production [14,48]. The cardoon flower produces a fruit that acts as a dispersal unit, generally called seeds. This is a common trait for cardoon and sunflower since they belong to the same botanical family (Asteraceae) [14,43,44,48,49]. Although constituting a small percentage of the biomass, the seeds have been widely studied due to their important bioactive properties and potential use for energy [14,18,39]. The seeds also have an important nutritional value, having high fat (23.7 g/100 g dry weight) and protein (30.4 g/100 g dry weight) contents. The cardoon seed oil has an interesting lipid profile, composed, on average, by 11% palmitic, 4% stearic, 25% oleic, and 60% linoleic fatty acids. The oil from cardoon can be used for food applications or the production of biodiesel [14,18,39].

#### 4.1.3. Paper Pulp

As cardoon can grow and flourish in hot and dry climate conditions and has high biomass productivity, its utilization in the production of paper pulp has been suggested [25,40]. Additionally, due to its similarities to eucalypt pulp, cardoon can be a good alternative in the hardwood pulp sector [14,25,45]. In a study by Gominho et al. [25], cardoon pulp was produced with good yields, with very little residual lignin, high bulk, and remarkable tensile strength properties. The authors concluded that the variables in production could be improved for even better results [25]. These results confirmed the potential of cardoon in the hardwood pulp sector, as it originated a good pulp with high yields, low rejection rate, and very good strength properties [25].

#### 4.1.4. Green Forage

As the cardoon crop may generate large amounts of biomass by-products when cultivated in large areas, it is important to find alternative ways to use them. Therefore, authors in this field have suggested the use of cardoon by-products as green forage for animal feed. Some authors have studied the nutrition value of green forage and have considered it safe and acceptable for animal feed [14,41,42,45,50]. Cajarville et al. [42,50] evaluated the nutrition value of cardoon for animal feed and concluded that it is a very good forage with good content in protein (156 g/kg dry matter), high digestibility coefficients (86.1 ± 1.3% for organic matter), high energy value (82.7 ± 1.6 MJ/kg dry matter), and suitability for ruminant feed. Cabiddu et al. [41] also evaluated cardoon seed pressed cakes to be used for the feeding of small ruminants. The authors concluded that, due to a high source of protein (18.52 ± 0.08% dry matter), fiber (1.22 ± 0.30%), phenolic compounds (32.7 ± 0.02 mg/tannic acid equivalent), and polyunsaturated fatty acid (PUFA), namely linoleic acid (1.7% dry matter) and oleic acid (0.7% dry matter), it is suitable for animal feed. The authors also suggested that the high content of phenolic compounds and PUFA of the green forage might be transferred to dairy products, increasing their nutritional value. It is important to develop more studies regarding this field because the utilization of crop biomass for animal feed can reduce the environmental problems associated with livestock production [51].

### 4.2. Source of Bioactive Compounds

#### 4.2.1. Stems

Stems and leaves, the most abundant waste regarding cardoon crops [16], may represent also a source of bioactive compounds. Studies concerning cardoon stems are scarce, but it has been identified as a source of caffeoylquinic acids [16,52,53]. Caffeoylquinic acids are natural antioxidants associated with the structural support of the plant since they establish bridges with the polymeric compounds of the cell wall [16,52]. Caffeoylquinic acids have been suggested to decrease the risk of chronic diseases including cancer and cardiovascular disease [6]. Pandino et al. [52] studied the cardoon stems and corroborated the richness in caffeoylquinic acids. Besides caffeoylquinic acids (17.7 g/kg dry matter), luteolin (3.0 g/kg dry matter) and apigenin (4.7 g/kg dry matter) were also identified in the cardoon stems. Several authors [53,54,55] also studied the globe artichoke stems and also concluded that they may represent a good source of caffeoylquinic acids. Globe artichoke stem presented, on average, a lower content of caffeoylquinic acids (13 g/kg dry matter) and lutein (0.4 g/kg dry matter), and apigenin was not detected [55]. Yet, the variability of the results may be attributed to genetic variation, crop management, post-harvest processing options, and environmental factors [6,53,54,55].

The stem presents good antioxidant activity, mainly due to its richness in bioactive compounds. More studies should be performed for a better knowledge of the total composition of phenolic compounds in cardoon stems.

#### 4.2.2. Leaves

Cardoon leaves represent, on average, about 60% of total cardoon waste [56]. Since the initial stage of the plants, the leaves showed beneficial properties, such as diuretic, hepato-protective, choleretic, hypocholesterolemic, anti-carcinogenic, and antibacterial effects [8,14,57,58]. Such properties are due to the high content in bioactive compounds presented by the leaves, such as chlorogenic acid, cynarine, and luteolin [8,16,59,60]. Cardoon leaves present also a high content of sesquiterpene lactones. The sesquiterpene lactones are responsible for the phytotoxic, cytotoxic, fungicidal, antiviral, and antimicrobial activity of cardoon. Thus, cardoon leaf extracts can be used in the development of herbicides of natural origin [61]. Cardoon leaves can as well be used in the preparation of alcoholic beverages as a flavoring agent [47].

## 5. Bioactive Properties of Cardoon Leaves

### 5.1. Nutritional Value

As far as the authors know, there is just one study regarding the nutritional value characterization of the leaves. The study indicates that cardoon leaves are a good source of carbohydrates and fat [59]. Cardoon leaves have an energetic contribution of 360.03 ± 1.09 kcal/100 g dry weight [59]. In terms of soluble sugars, cardoon leaves presented fructose, glucose, sucrose, and trehalose in its constitution [59]. For the organic acid profile, Chihoub et al. [59] identified oxalic, quinic, shikimic, citric, and fumaric acid in cardoon leaves. Oxalic acid was found to be the major organic acid, and fumaric acid was only found in trace amounts. The authors also studied the lipid profile of cardoon leaves and identified several fatty acids. The major fatty acid identified was α-linolenic acid (C18:3 n-3, PUFA) followed by linoleic acid (C18:2 n-6c, PUFA). According to the same study, cardoon leaves can be considered of high nutritional value as they have a high ratio of PUFA and saturated fatty acids (SFA) (PUFA/SFA = 3.80). Regarding the tocopherols, cardoon leaves present all four isoforms (α, β, γ, and δ), δ-tocopherol being the most abundant isoform. Tocopherols are known for their biological properties, for example, anti-inflammatory properties, and for their high antioxidant potential, when present in different isoforms [59].

### 5.2. Phenolic Composition and Antioxidant Properties

Several authors studied the antioxidant capacity of the cardoon leaves and stated the presence of polyphenolic compounds in high content [3,8,59,62]. Furthermore, traditionally leaves have been used in European medicine because of their known pharmacological properties, mainly due to the presence of cynarin and silymarin [14]. These compounds enhance liver and gallbladder function by stimulating the secretion of digestive juices [19].

So far, several polyphenol antioxidant compounds have been identified in cardoon leaves, chlorogenic acid and flavonoids being the most common ones [60]. Flavonoid presence in the leaves is suggested to be strategic due to its properties, since flavonoids are compounds that can absorb ultraviolet (UV) rays, specifically UV-B, so they can protect the plant from the harm that the radiation can cause [52,60]. Additionally, Pinelli et al. [60] compared the flavonoid content of cardoon that was and was not submitted to UV-light and concluded that the samples that were kept away from UV-light presented a lower number of flavonoids, confirming that flavonoids are essential to protect the plant from damage caused by UV rays. Besides, flavonoids have important antioxidant properties with important health benefits. Table 1 presents the main bioactive compounds that have been identified in cardoon leaves and their respective contents. Caffeoylquinic acid is the most common bioactive compound quantified in cardoon leaves. Pandino et al. [16] indicated that this compound possesses a potential inhibitory capacity of the development of cancers, exacerbated by the presence of reactive oxygen species. The bioactive content of cardoon leaves depends on several intrinsic and extrinsic factors such as genetic, environmental, handling, and storage, as well as the maturity of the plant [8,63,64]. Pandino et al. [65] studied the environmental effect on polyphenol content of the F1 population bred from a cross between a globe artichoke and a cultivated cardoon. The authors selected eight segregants that accumulated more caffeoylquinic acid in their leaves than did those of either of their parental genotypes. The selections were grown over two seasons to assess their polyphenol profile. The leaves of the first season had a higher content of polyphenols than those of the second season, considering that it rained less and had overall a higher average relative humidity. The authors concluded that the growing environment exerted a strong effect on polyphenol content.

These compounds have important properties in different fields of human wellbeing, namely antioxidant, antimicrobial, anti-inflammatory, anticancer, and anti-anxiety [36,52,58,59].

Several assays for measuring the antioxidant activity of the leaves or their potential to inhibit lipid oxidation can be performed. The ferric reducing-antioxidant power (FRAP) assay, DPPH free radical inhibition assay, β-carotene bleaching assay, thiobarbituric acid reactive substances (TBARS) inhibition, and the reducing power assay are some of these most commonly used techniques to evaluate the antioxidant activity either directly or indirectly. Numerous authors [8,52,59] executed these assays and confirmed the antioxidant activity of the cardoon leaf extracts [62] due to the presence of many bioactive compounds previously described. For example, Chihoub et al. [59] determined the antioxidant activity of cardoon leaf hydroethanolic extract (5 mg/mL) through the DPPH free radical inhibition assay (0.07 ± 0.00 mg/mL), reducing power (0.27 ± 0.09 mg/mL), β-carotene bleaching assay (0.19 ± 0.05 mg/mL), and TBARS inhibition (0.05 ± 0.01 mg/mL). The authors compared these results with cardoon leaf infusion preparations (DPPH free radical inhibition assay (0.22 ± 0.03 mg/mL), reducing power (0.22 ± 0.00 mg/mL), β-carotene bleaching assay (0.30 ± 0.04 mg/mL), and TBARS inhibition (0.40 ± 0.04 mg/mL)) and hydroethanolic extracts of turnip (DPPH free radical inhibition assay (1.57 ± 0.06 mg/mL), reducing power (1.07 ± 0.29 mg/mL), β-carotene bleaching assay (0.67 ± 0.15 mg/mL), and TBARS inhibition (0.60 ± 0.12 mg/mL)) and radish (DPPH free radical inhibition assay (0.14 ± 0.01 mg/mL), reducing power (0.58 ± 0.07 mg/mL), β-carotene bleaching assay (0.48 ± 0.02 mg/mL), and TBARS inhibition (0.43 ± 0.07 mg/mL)) and, assuming that lower results indicated higher antioxidant activity, concluded that cardoon leaf hydroethanolic extracts presented higher antioxidant activity.

### 5.3. Antimicrobial and Antifungal Properties

Cardoon leaves are known for having antimicrobial activity [8,63]. Falleh et al. [8] pointed out the antimicrobial activities of cardoon leaves due to their high content in phenolic compounds. The antimicrobial activities of cardoon leaves have been tested against several microorganisms of high importance for human beings due to their pathogenicity [8,63]. Cardoon leaves exhibit good antimicrobial activity against Gram-positive and Gram-negative bacteria, such as *Escherichia coli* and *Staphylococcus aureus*. However, they did not show antimicrobial activity against *Salmonella Typhimurium*. Table 2 features the studied species and their respective minimum inhibitory concentrations (MIC, which corresponds to the minimal extract concentration that inhibited the bacterial growth) or diameter of growth inhibition obtained by different authors. Scavo et al. [63] pointed out that Gram-positive bacteria are more susceptible than Gram-negative bacteria to plant extracts due to the permeability of the bacteria cell barrier, as well as the presence in the periplasmic space of enzymes that can break down molecules introduced from outside.

Authors have linked the antimicrobial activity of cardoon leaves to its richness in phenolic compounds, mainly due to the presence of lutein [8]. Nonetheless, more studies are needed for a better evaluation of the potential antimicrobial activities of cardoon leaves. Kukíc et al. [17] studied the antifungal activities of *C. cardunculus* involucral bract extracts, which showed potential antifungal activities. This may suggest that cardoon leaves may have antifungal activities too. Thus, more studies should be made to assess this issue.

### 5.4. Phytotoxic and Allelopathic Properties

Cardoon leaf extracts are also known for their allelopathic activities. Allelopathy is the direct or indirect effect of a plant over a target species (plants, algae, bacteria, or fungus) through the release of chemical compounds, allelochemicals, into the environment [61,67,68]. The allelochemicals can be integrated with weed management and can be introduced into the environment through volatilization from the aboveground parts of the plant, root exudation, foliar leaching, or plant residue decomposition [61,67,68,69,70]. Phenolic compounds and sesquiterpene lactones are the most common examples of allelochemicals and can be found in different parts of plants, such as leaves, roots, stems, rhizomes, seeds, flowers, and pollen [69,71]. Cardoon has been found to manifest phytotoxic activity on weeds and standard target species, and sesquiterpene lactones were identified as the most relevant allelochemicals [61,67,68,69,70,71,72]. Rial et al. [61] studied the potential phytotoxic activity of cardoon allelochemicals against the development of standard target species (lettuce, watercress, tomato, and onion) and weeds (barnyard grass and brachiaria). The authors used different solvents to perform cardoon extracts that were tested in phytotoxic bioassays, and the ethyl acetate extract had the highest inhibitory activity. The extract was very active on root growth in both standard target species and weeds, with values close to 80% inhibition in most species. The extract was also active on the germination and the shoot length of the cress. Moreover, the authors isolated six sesquiterpene lactones (aguerin B, grosheimin, 8α-acetoxyzaluzanin C, dehydromelitensin, cynaropicrin, and 11,13-dihydroxy-8-deoxy-grosheimin). Aguerin B, grosheimin, and cynaropicrin showed strong phytotoxicity against standard target species and weeds [61]. Other compounds such as caffeoylquinic acid, luteolin, and apigenin derivatives have been reported to show allelopathic activity against several crops [67,68,69,71]. More recently, studies by Kaab et al. [70] and Scavo et al. [69] confirmed that cardoon leaf extracts could present a suitable source of natural compounds for a good potential bioherbicide.

## 6. Cardoon Leaves and Potential Applications

Cardoon leaves are a source of several bioactive compounds with important health benefits. Therefore, it is important to exploit the potential uses of cardoon leaves for better use. Cardoon leaves, constituted by several bioactive compounds with antioxidant and antimicrobial activity, can be considered a potential ingredient in the food industry. The leaves could be used as a food additive or as an ingredient in the development of a novel food with functional properties and health benefits [9,73]. Plant extracts have been incorporated as an antioxidant in meat and meat products. The extracts inhibit lipid oxidation and meat degradation, which improved the nutritional quality and extended meat’ shelf life [74,75]. Thus, it can be foreseen that cardoon leaf extracts could also be incorporated into meat products to enhance their quality.

Another potential application of cardoon leaf is in the cosmetic industry. Several members of the Asteraceae family are used in the cosmetic industry for their bioactive compounds [76]. *Cynara scolymus* L., also known as globe artichoke, belongs to the Asteraceae family and is used in the cosmetic area for its richness in polyphenols content with antioxidant activity that can prevent aging and oxidative stress-related diseases and protect against UV-rays [77,78]. Due to its similarity, cardoon leaves may also be interesting to be used in this field.

Nonetheless, more studies should be made to assess the potential use of cardoon leaves in the food and cosmetics industries.

### Food Packaging

The primary function of food packaging is to protect foods during the transportation process until it reaches the final consumer, increasing the shelf life of food [79]. Mainly manufactured from non-biodegradable matrices, such as polyethylene, these packages act as a defense barrier against insects and microorganisms, some of which are pathogenic. They can also protect foods against possible impacts that may occur during the transportation process, as well as temperature variations and radiation [80,81].

Active and intelligent food packaging are relatively new packaging technologies that may increase even further food shelf life or help increase food quality. Intelligent food packaging is designed to monitor at least one condition of the packaged food, such as temperature (e.g., breaks in the cold chain) and leaks (e.g., leaks in vacuum packaging and modified atmosphere packaging). These packages normally have an indicator, visible to the consumer (e.g., color) that changes when the monitored condition reaches non-tolerable values [82].

Regarding active food packaging, although it is a relatively new concept, it was inspired by the “packages” used by human civilization in ancient times, where the primitive man kept his food in leaves, which were composed of active compounds and that could help to increase the shelf life of food. Nowadays, there are two types of active food packaging. The absorption/adsorption active packaging absorb/adsorb possible deterioration gases and liquids that are generated through the natural food’s degradation process [83,84]. The emission active packaging directly interacts with foods, emitting substances or compounds to the packaged foods. Normally, these substances or compounds have powerful antioxidant and antimicrobial activities that will help retard the food’s degradation process, thus increasing the shelf life of food [84,85,86].

Food additives also help to delay natural food degradation. Normally, these substances are applied directly into foods, and their application is regulated by the Food and Drug Administration in the United States of America and through the Regulation no. 1333/2008 and its amendments in the European Union [87,88]. A food additive is a substance, not usually consumed as a food itself, added to foods for technological purposes [87]. Although their applications and limits are highly controlled by the respective institutes and entities, their long-term effects on human health are still unknown. Actually, in recent years, some synthetic food antioxidants, such as butylated hydroxytoluene (BHT) and butylated hydroxyanisole (BHA) have been associated with the promotion of carcinogenesis and the emergence of neurodegenerative diseases, such as Parkinson’s and Alzheimer’s diseases [89,90,91]. That being said, active food packaging can help decrease the applied quantities of these compounds and, consequently, the consumer’s ingestion of these compounds. Additionally, the investigation and application of new natural compounds from food by-products can be an answer to this problem, as these by-products are rich in active compounds, with powerful antimicrobial, antifungal, and antioxidant activities.

So far, plant extracts and plant essential oils with known antioxidant and antimicrobial activity have been used in food packaging to control lipid oxidation and microbial deterioration [79,83,92,93,94,95]. For instance, Rizzo et al. [96] conducted a study on globe artichoke slices to evaluate dipping in locust bean gum edible coating with *Foeniculum vulgare* essential oil to extend its shelf life. The locust bean gum edible coating with added *Foeniculum vulgare* essential oil effectively decreased all microbiological groups tested and improved physical, chemical, and sensory qualities during 11 days of storage. Mazzaglia et al. [97] studied the effect of cardoon extract in reducing microbiological contamination and increasing the shelf life of aubergine-based burgers. Two concentrations of extract were used (1% and 3%) for the preparation of burgers, and the microbial load and sensory changes of vacuum-packed burgers were analyzed at processing day and after 30 and 105 days of storage. The burger with 3% of cardoon extract presented the best results, as it significantly reduced the growth of bacteria up to 30 and 105 days of refrigerated storage. Taking this into account, and knowing the bioactive characterization of cardoon leaf extract, it can present itself as a potential candidate in the production of active packaging. Therefore, studies should be done following this perspective.

## 7. Conclusion and Future Perspectives

Recent studies have indicated that cardoon leaves are rich in several polyphenol compounds, with several health benefits. Additionally, cardoon leaves are pointed out to have potential antimicrobial activity. Caffeoylquinic acids, which are the major bioactive compounds identified in cardoon leaves, are naturally-occurring antioxidant compounds that have been suggested for use as natural additives for extending the shelf life of food products.

Moreover, as leaves are considered cardoon by-products, they can have economic benefits if their natural antioxidants, with benefits to human health, are extracted and applied in food packaging to increase shelf life.

Nevertheless, cardoon by-products and their potential for application in several industrial fields such as cosmetics, food, and food packaging is still not entirely known and should be investigated further for a better comprehension of the potential uses of this valuable Mediterranean crop.

## Figures and Tables

**Table 1:** Main bioactive compounds of cardoon leaves.

Species/Variety	Presentation form and Extraction Procedure	Main Bioactive Compounds and Levels Found	Main Conclusion	Reference
*C. cardunculus* L. var. *sylvestris*	Hydroethanolic extract: 1 g of dried samples was added to 30 mL of solvent (ethanol:water, 80:20, *v/v*) for 1 h. It was filtered and reextracted in 30 mL of solvent for 1 h. The combined extracts were evaporated until dryness of the ethanol at 35 °C and the aqueous phase was frozen and lyophilized.	3-O-Caffeoylquinic acid (0.48 ± 0.01 mg/g of extract)	The authors concluded that the hydroethanolic was more effective than the infusion preparation in the extraction of active compounds.	[59]
		5-Hydroxyferuloylglycoside (1.3 ± 0.1 mg/g of extract)		
		4-O-Caffeoylquinic acid (13.6 ± 0.1 mg/g of extract)		
		5-O-Caffeoylquinic acid (0.179 ± 0.001 mg/g of extract)		
		3-O-Feruloylquinic acid (0.32 ± 0.01 mg/g of extract)		
		5-O-Feruloylquinic acid (0.53 ± 0.01 mg/g of extract)		
		Luteolin-O-hexoside (5.5 ± 0.1 mg/g of extract)		
		Pinoresinol-O-hexoside (0.37 ± 0.01 mg/g of extract)		
		3,4-O-Dicaffeoylquinic acid (6.492 ± 0.002 mg/g of extract)		
		3,5-O-Dicaffeoylquinic acid (0.331 ± 0.001 mg/g of extract)		
		Luteolin-O-malonylhexoside (7.39 ± 0.02 mg/g of extract)		
		Acetylapigenin-O-hexoside (1.85 ± 0.01 mg/g of extract)		
		Total phenolic acids (23.6 ± 0.2 mg/g of extract)		
		Total flavonoids (14.7 ± 0.1 mg/g of extract)		
		Total phenolic compounds (38.3 ± 0.3 mg/g of extract)		
	Infusion preparation: 1 g of dried samples was added to 100 mL of boiling distilled water, left to stand for 5 min, filtered, and then frozen and lyophilized.	3-O-Caffeoylquinic acid (0.66 ± 0.01 mg/g of extract)		
		5-Hydroxyferuloylglycoside (0.95 ± 0.01 mg/g of extract)		
		4-O-Caffeoylquinic acid (10.2 ± 0.1 mg/g of extract)		
		5-O-Caffeoylquinic acid (0.185 ± 0.003 mg/g of extract)		
		3-O-Feruloylquinic acid (0.25 ± 0.1 mg/g of extract)		
		5-O-Feruloylquinic acid (0.39 ± 0.01 mg/g of extract)		
		Luteolin-O-hexoside (1.6 ± 0.1 mg/g of extract)		
		3,4-O-Dicaffeoylquinic acid (3.5 ± 0.2 mg/g of extract)		
		3,5-O-Dicaffeoylquinic acid (0.319 ± 0.001 mg/g of extract)		
		Luteolin-O-malonylhexoside (5.8 ± 0.2 mg/g of extract)		
		Acetylapigenin-O-hexoside (4.73 ± 0.04 mg/g of extract)		
		Total phenolic acids (16.0 ± 0.3 mg/g of extract)		
		Total flavonoids (12.1 ± 0.2 mg/g of extract)		
		Total phenolic compounds (29 ± 1 mg/g of extract)		
*C. cardunculus* L. var. *sylvestris*	Dried samples were extracted in 1 mL of 70% methanol, containing butylated hydroxytoluene and hesperetin, for 1 h at room temperature with shaking. After centrifugation, the supernatant was transferred to a microfuge tube, and the sample was centrifuged once more with 0.25 mL of 70% methanol. The supernatants were combined and kept at −20 °C until analysis.	Luteolin glucoside (0.8 g/kg DM)	Both wild and cultivated cardoon presented a good profile of polyphenols, but the cultivated cardoon profile was richer and more variable than the wild cardoon. The apigenin derivates were the most abundant compounds in both cases.	[52]
		Luteolin glucuronide (1.9 ± 0.1 g/kg DM)		
		Luteolin (0.3 g/kg DM)		
		Total luteolin (3.2 g/kg DM)		
		Apigenin rutinoside (0.2 g/kg DM)		
		Apigenin glucuronide (3.3 ± 0.1 g/kg DM)		
		Apigenin (1.3 g/kg DM)		
		Total apigenin (4.8 g/kg DM)		
		Total measured polyphenols (8.0 g/kg DM)		
*C. cardunculus* L. var. *altilis*		5-Caffeoylquinic acid (0.3 g/kg DM)		
		Total caffeoylquinic acid (0.3 g/kg DM)		
		Luteolin glucoside (0.1 g/kg DM)		
		Luteolin glucuronide (2.4 ± 0.3 g/kg DM)		
		Luteolin (0.9 ± 0.1 g/kg DM)		
		Total luteolin (3.4 g/kg DM)		
		Apigenin rutinoside (0.5 g/kg DM)		
		Apigenin glucuronide (3.3 ± 0.3 g/kg DM)		
		Apigenin malonylglucoside (0.4 g/kg DM)		
		Apigenin (1.3 ± 0.2 g/kg DM)		
		Total apigenin (5.6 g/kg DM)		
		Total measured polyphenols (9.3 g/kg DM)		
*C. cardunculus*	Leaves were dried at room temperature for two weeks. The extract was made with 2.5 g of dry powder with 25 mL of solvent (methanol) under stirring for 30 min. Then the extract was filtered, evaporated to dryness under vacuum, and stored at 4 °C until analysis.	Polyphenol content (14.79 (mg/GAE g DW)	The sample exhibit a very good bioactive compounds profile, greater than several other species, mainly due to the hard climate conditions of development.	[8]
		Flavonoid content (9.08 mg/CE g DW)		
		Tannin content (1.96 mg/CE g DW)		
*C. cardunculus* L. var. *sylvestris*	The samples were lyophilized, after comminuted to a powder. The powder was extracted in quadruplicate with 3 × 50 mL of ethanol (70% *v/v*) at room temperature, under stirring. The extracts were completely defatted with n-hexane (4 × 20 mL), then concentrated under vacuum and rinsed with the extraction solvent to a final volume of 25 mL. Hydro-alcoholic extracts were stored at −20 °C until use.	1-O-Caffeoylquinic acid (8.23 ± 0.68 µmol/g d wt)	Both wild and cultivated cardoon presented a good profile of polyphenols, but the wild cardoon profile was richer and more variable than the cultivated cardoon. The samples were both rich in different flavonoids compounds, and the authors emphasized their role in the protection of the plant against UV-radiation.	[60]
		Chlorogenic acid (61.84 ± 2.09 µmol/g d wt)		
		Luteolin 7-O-rutinoside (1.10 ± 0.21 µmol/g d wt)		
		Luteolin 7-O-glucoside (27.29 ± 1.87 µmol/g d wt)		
		Dicaffeoylquinic acids (114.57 ± 4.25 µmol/g d wt)		
		Luteolin 7-O-malonylglucoside (14.62 ± 0.41 µmol/g d wt)		
		Apigenin 7-O-rutinoside (1.11 ± 0.11 µmol/g d wt)		
		Luteolin (0.80 ± 0.01 µmol/g d wt)		
		Luteolin 7-O-glucuronide (34.61 ± 1.48 µmol/g d wt)		
		Apigenin 7-O-glucuronide (23.72 ± 0.06 µmol/g d wt)		
		Apigenin (4.74 ± 0.14 µmol/g d wt)		
		Total polyphenols (292.63 µmol/g d wt)		
*C. cardunculus* L. var. *altilis*		1-O-Caffeoylquinic acid (9.53 ± 2.63 µmol/g d wt)		
		Chlorogenic acid (73.68 ± 4.83 µmol/g d wt)		
		Luteolin 7-O-glucoside (33.55 ± 8.21 µmol/g d wt)		
		Dicaffeoylquinic acids (29.17 ± 9.26 µmol/g d wt)		
		Succinyldicaffeoylquinic acid (8.67 ± 0.41 µmol/g d wt)		
		Luteolin 7-O-malonylglucoside (43.00 ± 0.50 µmol/g d wt)		
		Succinyldicaffeoylquinic acid (2.20 ± 0.16 µmol/g d wt)		
		Luteolin (1.68 ± 0.08 µmol/g d wt)		
		Luteolin 7-O-glucuronide (13.70 ± 2.40 µmol/g d wt)		
		Total polyphenols (215.18 µmol/g d wt)		
*C. cardunculus* L. var. *scolymus*	Infusion: 20 g of dried chopped leaves was added to 1000 mL of ultra-pure water at 95 °C, and the mixture was left to stand for 10 min and then filtered through cotton. Extract were frozen and freeze-dried.	Chlorogenic acid (64 ± 2 mg/g extract)	All extracts presented good phenolic content. The infusion extract presented higher phenolic content. Chlorogenic acid was the major phenolic compound identified in all extracts.	[66]
		*p*-Coumaroylquinic acid (1.1 ± 0.1 mg/g extract)		
		5-Feruloylquinic acid (1.6 ± 0.3 mg/g extract)		
		Luteolin-7-rutinoside (7.6 ± 0.1 mg/g extract)		
		Luteolin-7-glucoside (cynaroside) (3.0 ± 0.1 mg/g extract)		
		3,4-Dicaffeoylquinic acid (2.1 ± 0.1 mg/g extract)		
		1,3-Dicaffeoylquinic acid (cynarin) (22.4 ± 0.1 mg/g extract)		
		Luteolin-7-malonyl-hexoside (1.7 ± 0.1 mg/g extract)		
		4,5-Dicaffeoylquinic acid (5.1 ± 0.1 mg/g extract)		
		Phenolic contents (108 ± 2 mg/g extract)		
	Decoction: The dried chopped leaves (20 g) were added to 1000 mL of ultrapure water, heated, and boiled for 10 min, and then the mixture was removed from the heat and left to stand for 5 min to be filtered through cotton. The extract was frozen and freeze-dried.	Chlorogenic acid (40 ± 3 mg/g extract)		
		*p*-Coumaroylquinic acid (1.1 ± 0.1 mg/g extract)		
		5-Feruloylquinic acid (0.9 ± 0.1 mg/g extract)		
		Luteolin-7-rutinoside (7.4 ± 0.8 mg/g extract)		
		Luteolin-7-glucoside (cynaroside) (2.9 ± 0.4 mg/g extract)		
		3,4-Dicaffeoylquinic acid (0.9 ± 0.3 mg/g extract)		
		1,3-Dicaffeoylquinic acid (cynarin) (6.5 ± 0.5 mg/g extract)		
		Luteolin-7-malonyl-hexoside (1.3 ± 0.1 mg/g extract)		
		4,5-Dicaffeoylquinic acid (1.9 ± 0.4 mg/g extract)		
		Phenolic content (63 ± 5 mg/g extract)		
	Hydroalcoholic extract: 20 g of dried chopped leaves was added to 1000 mL of a mixture of ethanol/water (70:30, *v/v*) and stirred on an orbital shaker (70 rpm) for 12 h at 25 °C. The hydroalcoholic mixture was filtered through cotton, concentrated under reduced pressure in a rotary evaporator (40 °C), and then freeze-dried.	Chlorogenic acid (43 ± 2 mg/g extract)		
		5-Feruloylquinic acid (0.6 ± 0.1 mg/g extract)		
		Luteolin-7-rutinoside (9.3 ± 0.4 mg/g extract)		
		Luteolin-7-glucoside (cynaroside) (3.8 ± 0.3 mg/g extract)		
		3,4-Dicaffeoylquinic acid (0.03 ± 0.01 mg/g extract)		
		1,3-Dicaffeoylquinic acid (cynarin) (14 ± 1 mg/g extract)		
		Luteolin-7-malonyl-hexoside (1.0 ± 0.1 mg/g extract)		
		4,5-Dicaffeoylquinic acid (1.1 ± 0.1 mg/g extract)		
		Phenolic content (73 ± 4 mg/g extract)		
*C. cardunculus* L. var. *altilis*	Bidistilled water extract: Dried leaves were soaked in bidistilled water in the ratio 1:10 *w/v*. Then, the mixture was kept under dark conditions for 72 h at room temperature (20 °C ± 1) and filtered to eliminate the solid fraction.	5-O-caffeoylquinic acid (51.3 ± 0.2 mg/L)	Caffeoylquinic acid represents more than 50% of the total phenolic compounds present in the extracts. The methanolic extract was more efficient in extracting the compounds, followed by the ethanolic and water extracts.	[63]
		1,5-O-dicaffeoylquinic acid (119.3 ± 33.3 mg/L)		
		Monosuccinildicaffeoylquinic acid (37.6 ± 1.0 mg/L)		
		Total caffeoylquinic acid (208 mg/L)		
		Luteolin 7-O-glucoronide (10.9 ± 0.4 mg/L)		
		Luteolin (53.2 ± 0.4 mg/L)		
		Total luteolin (64 mg/L)		
		Cynaropicrin (5.4 ± 0.2 mg/L)		
		Total measured polyphenols (272 mg/L)		
	Ethanolic extract: Dried leaves were soaked in 80% ethanol in the ratio 1:10 *w/v*. Then, the mixture was kept under dark conditions for 72 h at room temperature (20 °C ± 1) and filtered to eliminate the solid fraction. The ethanolic solution was evaporated at 35 °C with a rotary evaporator, and the residue was dissolved in bidistilled water to maintain the same ratio.	5-O-caffeoylquinic acid (340.0 ± 0.9 mg/L)		
		1,5-O-dicaffeoylquinic acid (230.5 ± 1.5 mg/L)		
		Monosuccinildicaffeoylquinic acid (36.4 ± 0.3 mg/L)		
		Total caffeoylquinic acid (607 mg/L)		
		Luteolin 7-O-glucoronide (189.4 ± 0.06 mg/L)		
		Luteolin 7-O-malonylglucoside (50.5 ± 0.01 mg/L)		
		Total luteolin (240 mg/L)		
		Apigenin 7-O-glucoside (45.4 ± 0.4 mg/L)		
		Apigenin 7-O-glucoronide (87.7 ± 0.8 mg/L)		
		Apigenin malonylglucoside (62.0 ± 1.6 mg/L)		
		Apigenin (3.8 ± 0.1 mg/L)		
		Total apigenin (199 mg/L)		
		Cynaropicrin (10.7 ± 0.9 mg/L)		
		Total measured polyphenols (1046 mg/L)		
*C. cardunculus* L. var. *altilis*	Methanolic extract: Dried leaves were soaked in 70% methanol in the ratio 1:10 *w/v*. Then, the mixture was kept under dark conditions for 72 h at room temperature (20 °C ± 1) and filtered to eliminate the solid fraction. The methanolic solution was evaporated at 35 °C with a rotary evaporator, and the residue was dissolved in bidistilled water to maintain the same ratio.	5-O-caffeoylquinic acid (632.0 ± 0.1 mg/L)	Caffeoylquinic acid represents more than 50% of the total phenolic compounds present in the extracts. The methanolic extract was more efficient in extracting the compounds, followed by the ethanolic and water extract.	[63]
		1,5-O-dicaffeoylquinic acid (206.4 ± 0.3 mg/L)		
		Monosuccinildicaffeoylquinic acid (59.4 ± 0.01 mg/L)		
		Total caffeoylquinic acid (898 mg/L)		
		Luteolin 7-O-glucoronide (22.7 mg/L)		
		Luteolin 7-O-malonylglucoside (83.3 ± 0.01 mg/L)		
		Total luteolin (106 mg/L)		
		Apigenin 7-O-glucoside (61.7 ± 0.04 mg/L)		
		Apigenin 7-O-glucoronide (115.0 ± 0.2 mg/L)		
		Apigenin malonylglucoside (72.1 ± 0.04 mg/L)		
		Total apigenin (249 mg/L)		
		Cynaropicrin (15.8 ± 0.1 mg/L)		
		Total measured polyphenols (1253 mg/L)		

CE—catechin equivalent; DM—dry matter; DW—dry weight; GAE—gallic acid equivalent.

**Table 2:** Main antimicrobial activity of cardoon leaves.

Species/Variety	Presentation form and Extraction Procedure	Main Bioactive Compounds and Levels Found	Main Conclusion	Reference
*C. cardunculus* L. var. *sylvestris*	Hydroethanolic extract: 1 g of dried samples in 30 mL of solvent (ethanol:water, 80:20, *v/v*) for 1 h. Filtered and re-extracted in 30 mL of solvent for 1 h. The combined extracts were evaporated to dryness of the ethanol at 35 °C, and the aqueous phase was frozen and lyophilized.	*Escherichia coli* (MIC = 2.5 mg/mL)	Both extracts were demonstrated to have good antimicrobial activities, with low MIC values. The authors’ concluded that the ethanolic extract was more effective when compared with other results obtained in this study.	[59]
		*Escherichia coli* ESBL (MIC = 10 mg/mL)		
		*Klebsiella pneumoniae* (MIC = 20 mg/mL)		
		*Klebsiella pneumoniae* ESBL (MIC = 20 mg/mL)		
		*Morganella morganii* (MIC = 10 mg/mL)		
		*Pseudomonas aeruginosa* (MIC = 10 mg/mL)		
		*Enterococcus faecalis* (MIC = 5 mg/mL)		
		*Listeria monocytogenes* (MIC = 10 mg/mL)		
		MRSA (MIC = 5 mg/mL)		
		MSSA (MIC = 5 mg/mL)		
	Infusion preparation: 1 g of dried samples was added to 100 mL of boiling distilled water, left to stand for 5 min, filtered, and then frozen and lyophilized.	*Escherichia coli* (MIC = 2.5 mg/mL)		
		*Escherichia coli* ESBL (MIC = 5 mg/mL)		
		*Klebsiella pneumoniae* (MIC = 20 mg/mL)		
		*Klebsiella pneumoniae* ESBL (MIC = 20 mg/mL)		
		*Morganella morganii* (MIC = 2.5 mg/mL)		
		*Pseudomonas aeruginosa* (MIC = 20 mg/mL)		
		*Enterococcus faecalis* (MIC = 10 mg/mL)		
		*Listeria monocytogenes* (MIC = 10 mg/mL)		
		MRSA (MIC = 5 mg/mL)		
		MSSA (MIC = 5 mg/mL)		
*C. cardunculus*	Extract: Leaves were dried at room temperature for two weeks. The extract was made with 2.5 g of dry powder with 25 mL of solvent (methanol), under stirring for 30 min. Then the extract was filtered and evaporated to dryness under vacuum and stored at 4 °C until analysis.	*Staphylococcus aureus* ATCC25923 (DGI = 25.7 ± 0.6 mm)	The extract was effective against several human pathogenic bacteria but unfortunately had no activity against *Salmonella typhimurium* LT2. Antimicrobial activities could be related to the presence of phenolic compounds.	[8]
		*Staphylococcus epidermidis* CIP106510 (DGI = 20.3 mm)		
		*Micrococcus luteus NCIMB 8166 (DGI = 21.7 ± 0.6 mm)*		
		*Escherichia coli* ATCC 35,218 (DGI = 22.3 mm)		
		*Enterococcus faecalis* ATCC29212 (DGI = 16.3 ± 0.6 mm)		
		*Listeria monocytogenes* ATCC19115 (DGI = 9.3 ± 0.6 mm)		
		*Pseudomonas aeruginosa* ATCC 27,853 (DGI = 13.7 ± 0.6 mm)		
		*Salmonella typhimurium* LT2 (DGI = 0 mm)		
*C. cardunculus* L. var. *altilis*	Bidistilled water extract: Dried leaves were soaked in bidistilled water in the ratio 1:10 *w/v*. Then, the mixture was kept under dark conditions for 72 h at room temperature (20 °C ± 1) and filtered to eliminate the solid fraction.	*Bacillus cereus* (DGI = 0.7 cm)	Water extract was effective against Gram-positive bacteria, although methanolic and ethanolic extracts controlled the growth more effectively. Regarding Gram-negative bacteria, the methanolic extract was not effective, and the ethanolic extract showed detectable antibacterial activity. Overall, the ethanolic extract was more efficient against the studied bacteria when compared to the other two extracts.	[63]
		*Bacillus megaterium* (DGI = 0.8 ± 0.1 cm)		
		*Listeria innocua* (DGI = 0.8 cm)		
		*Pseudomonas syringae pv*. Tomato (DGI = 1.2 cm)		
		*Rhodococcus fascians* (DGI = 0.6 cm)		
		*Staphylococcus aureus* (DGI = 0.7 cm)		
		*Xanthomonas perforans* (DGI = 1.5 ± 0.1 cm)		
	Ethanolic extract: Dried leaves were soaked in ethanol 80% in the ratio 1:10 *w/v*. Then, the mixture was kept under dark conditions for 72 h at room temperature (20 °C ± 1) and filtered to eliminate the solid fraction. The ethanolic solution was evaporated at 35 °C with a rotary evaporator, and the residue was dissolved in bidistilled water to maintain the same ratio.	*Bacillus cereus* (DGI = 0.9 ± 0.1 cm)		
		*Bacillus megaterium* (DGI = 2.3 ± 0.1 cm)		
		*Bacillus subtilis* (DGI = 0.8 cm)		
		*Listeria innocua* (DGI = 0.8 ± 0.1 cm)		
		*Pseudomonas fluorescens* (DGI = 0.7 ± 0.1 cm)		
		*Pseudomonas syringae pv*. Tomato (DGI = 0.6 cm)		
		*Rhodococcus fascians* (DGI = 1.2 ± 0.1 cm)		
		*Staphylococcus aureus* (DGI = 1.1 ± 0.1 cm)		
		*Xanthomonas perforans* (DGI = 0.4 cm)		
	Methanolic extract: Dried leaves were soaked in methanol 70% in the ratio 1:10 *w/v*. Then, the mixture was kept under dark conditions for 72 h at room temperature (20 °C ± 1) and filtered to eliminate the solid fraction. The methanolic solution was evaporated at 35 °C with a rotary evaporator and the residue was dissolved in bidistilled water to maintain the same ratio.	*Bacillus cereus* (DGI = 1.3 ± 0.1 cm)		
		*Bacillus megaterium* (DGI = 1.3 ± 0.1 cm)		
		*Bacillus subtilis* (DGI = 0.8 cm)		
		*Listeria innocua* (DGI = 1.2 cm)		
		*Rhodococcus fascians* (DGI = 1.2 cm)		
		*Staphylococcus aureus* (DGI = 1 ± 0.1 cm)		

DGI—diameter of growth inhibition; ESBL—extended spectrum β-lactamases; MIC values correspond to the minimal extract concentration that inhibited the bacterial growth; MRSA—methicillin-resistant *Staphylococcus aureus*; MSSA—methicillin-susceptible *Staphylococcus aureus*.
